# Blood cell count indexes as predictors of anastomotic leakage in elective colorectal surgery: a multicenter study on 1432 patients

**DOI:** 10.1186/s12957-020-01856-1

**Published:** 2020-05-06

**Authors:** Panagiotis Paliogiannis, Simona Deidda, Svilen Maslyankov, Tsvetelina Paycheva, Ahmed Farag, Abdrabou Mashhour, Evangelos Misiakos, Dimitrios Papakonstantinou, Michal Mik, Joanna Losinska, Fabrizio Scognamillo, Fabio Sanna, Claudio Francesco Feo, Giuseppe Cherchi, Andreas Xidas, Angelo Zinellu, Angelo Restivo, Luigi Zorcolo

**Affiliations:** 1 Experimental Pathology and Oncology, Department of Medical, Surgical and Experimental Sciences, University of Sassari, Viale San Pietro 43, 07100 Sassari, Italy; 2 Colorectal Surgery Unit, Department of Surgical Sciences, University of Cagliari, Monserrato, 09042 Cagliari, Italy; 3 Second Surgery Clinic, Department of Surgery, Medical University of Sofia, G.Sofijski str. 1, 1404 Sofia, Bulgaria; 4 Colorectal Surgery Unit, General Surgery Department, Faculty of Medicine, Cairo University, Kaser Alainy Hospital, 89 Almanial, Cairo, Egypt; 5 Third Department of Surgery, Attikon University Hospital, Medical School, National and Kapodistrian University of Athens, 12462 Athens, Greece; 6 Department of General and Colorectal Surgery, Medical University of Lodz, Plac Hallera 1, 90-647 Lodz, Poland; 7 First Surgery Clinic, Department of Medical, Surgical and Experimental Sciences, University of Sassari, Viale San Pietro 43, 07100 Sassari, Italy; 8 Second Surgery Clinic, Department of Medical, Surgical and Experimental Sciences, University of Sassari, Viale San Pietro 43, 07100 Sassari, Italy; 9 Unit of Surgery, Nostra Signora della Mercede Hospital of Lanusei, Via Ospedale, 08045 Lanusei, Italy; 10 Clinical Biochemistry and Clinical Molecular Biology, Department of Biomedical Sciences, University of Sassari, Viale San Pietro 43, 07100 Sassari, Italy

**Keywords:** Colorectal surgery, Anastomotic leakage, NLR, PLR, LMR

## Abstract

**Background:**

The aim of this study was to evaluate a series of blood count inflammation indexes in predicting anastomotic leakage (AL) in elective colorectal surgery.

**Methods:**

Demographic, pathologic, and clinical data of 1432 consecutive patients submitted to colorectal surgery in eight surgical centers were retrospectively evaluated. The neutrophil to lymphocyte (NLR), derived neutrophil to lymphocyte (dNLR), lymphocyte to monocyte (LMR), and platelet to lymphocyte (PLR) ratios were calculated before surgery and on the 1st and 4th postoperative days, in patients with or without AL.

**Results:**

There were 106 patients with AL (65 males, mean age 67.4 years). The NLR, dNLR, and PLR were significantly higher in patients with AL in comparison to those without, on both the 1st and 4th postoperative days, but significance was greater on the 4th postoperative day. An NLR cutoff value of 7.1 on this day showed the best area under the curve (AUC 0.744; 95% CI 0.719–0.768) in predicting AL.

**Conclusions:**

Among the blood cell indexes of inflammation evaluated, NLR on the 4th postoperative day showed the best ability to predict AL. NLR is a low cost, easy to perform, and widely available index, which might be potentially used in clinical practice as a predictor of AL in patients undergoing elective colorectal surgery.

## Background

Anastomotic leakage (AL) is one of the most severe complications in modern colorectal surgery. It has been reported to occur in 3 to 27% of patients in relation to specific risk factors, despite recent improvements in the preoperative selection and preparation of the patients, the evolution of minimally invasive surgical techniques, the stapling devices used to fashion the anastomoses, and the postoperative management of the patients [1, 2]. Anastomotic leakage represents the most common cause of unplanned reoperation in large colorectal surgery cohorts [3]. Furthermore, AL is associated with greater short-term mortality, poorer oncological outcomes and overall survival, as well as higher costs for healthcare systems [4–6].

Anastomotic leakage can manifest clinically in several ways, in relation to the grade of the anastomotic breakdown, the anatomical site (colon or rectum) and the type of the surgical procedure, the general condition of the patient, and the presence of a protective stoma. Generally, early AL is caused by technical errors or defects, has a major clinical impact, and reoperation is often needed to treat it. Most commonly, AL occurs between the 5th and 8th postoperative days, and has a variable clinical presentation; minor leaks can be treated conservatively using drains to evacuate possible infectious collections, while major defects require re-intervention to clean the abdomen and restore the intestinal integrity or exteriorize the bowel [7]. In all patients, a prompt diagnosis is crucial because a delay in antibiotic administration from the onset of septic shock has been associated with a decrease in survival of 7.6% per hour [8]. The discovery of biomarkers able to predict AL early after colorectal surgery would bring consistent advantages in the management and outcomes of this complication.

To this regard, several biomarkers have been evaluated so far, most of them related to the inflammatory response to surgical manipulation, and the consequent reparative events in resected tissues. Factors like interleukins, C-reactive protein (CRP), procalcitonin (PCT), Na^+^, tissue plasminogen activator, and soluble fibrin have been evaluated in blood samples, as well as indexes including the cells participating in the inflammatory process, like the neutrophil to lymphocyte ratio (NLR) [9, 10]. The latter has been demonstrated to be a prognostic factor in numerous diseases, including primary and metastatic colorectal cancer [11–14]. Furthermore, it has been associated with the outcomes of several types of surgical procedures, and with the onset of postoperative complications [15–19]. However, poor data are currently available regarding its role in predicting AL in colorectal surgery. In the present study, we investigated the role of NLR and derived neutrophil to lymphocyte ratio (dNLR) as predictive markers of AL, along with the role of the lymphocyte to monocyte (LMR) and the platelet to lymphocyte (PLR) ratios, in 1432 patients with colorectal cancer who underwent elective surgical resection in eight different centers.

## Methods

Data of consecutive patients with histologically proven colorectal cancer, and undergoing elective surgery at the surgical units involved in the study from January 1, 2013 through December 31, 2017 were collected in an electronic database. Demographic and clinical data, including sex, age, body mass index (BMI), American Society of Anesthesiologists (ASA) score, Charlson comorbidity index, localization and histology of the disease, as well as the stage of the disease according to the American Joint Committee on Cancer (AJCC) staging system (7th edition), were registered. Tumor distance from the anocutaneous line was included for patients with rectal cancer. Furthermore, details regarding the surgical procedure, postoperative course, morbidity, and 30-day mortality were collected.

The inclusion criteria were as follows: (a) patients with histologically proven colorectal cancer; (b) patients undergoing elective surgical procedure with an open or laparoscopic approach; (c) patients with available clinical, surgical, and pathological data; (d) patients with available blood cell counts before surgery, and at the 1st and 4th postoperative days; and (e) patients who signed an informed consent for each procedure performed. The exclusion criteria were the following: (a) patients younger than 18, (b) those operated on an emergency setting, and (c) those who did not have an anastomosis. Patients who had a clinically manifested anastomotic leakage (Extended Clavien-Dindo classification stage III through V) [20] within 30 days from surgery were included in the AL group; when necessary, AL was confirmed by imaging or endoscopic techniques. All the operations were performed by senior surgeons, and the anastomoses were made up hand-sewn or with stapling devices; the choice of the technique or the stapling device was made by the surgeon based on the localization of the disease, the anatomical conditions of the patients, and his/her experience. The study was carried out in accordance with the principles of the Declaration of Helsinki, and was approved by the ethics committee of University Hospital (A.O.U.) of Cagliari (Italy).

Regarding laboratory tests, fasting blood samples were obtained with standard procedures and methodologies dictated by the current international and national guidelines, adopted by the institutions involved in the study; the samples were processed and analyzed in certified laboratories. Complete blood counts before the operation, on the 1st postoperative day, and on the 4th postoperative day were retrieved, and the NLR, dNLR (neutrophils/white blood cells—neutrophils), LMR, and PLR were calculated.

All results were expressed as mean (mean ± SD) or median values (median and IQR). Variable distribution was assessed by the Shapiro-Wilcoxon test. Statistical differences between groups were compared using unpaired Student’s *t* test or Mann-Whitney rank sum test, as appropriate. Correlations between variables were assessed by Pearson’s correlation or Spearman’s correlation, as appropriate. Multiple comparisons were performed by one-way ANOVA, student-Newman-Keuls test or Kruskal-Wallis test, as appropriate. Levene’s test for equality of error variances was employed. Logistic regression analysis was employed to investigate the association of NLR and other risk factors with AL.

The ability of the studied parameters to predict AL was analyzed using receiver operating characteristic (ROC) curve analysis. Optimal cutoff maximizing sensitivity and specificity was selected. Sensitivity and specificity were reported using the optimal ROC curve value according to the Youden index. The results of the area under the curve (AUC) represented the global accuracy of the tests performed, 0.91–1.00 (excellent), 0.81–0.90 (good), 0.71–0.80 (fair), 0.61–0.70 (poor), and 0.51–0.60 (fail). Statistical analyses were performed using MedCalc for Windows, version 15.4 64 bit (MedCalc Software, Ostend, Belgium) and SPSS for Windows, version 14.0 32 bit (IBM Corporation; Armonk, NY, USA).

## Results

The global number of patients enrolled in the study was 1432; among them, 817 (57%) were male, and the mean age was 65.8 (± 13.7). Globally, 106 (7.4%) patients with AL fulfilling the selection criteria were registered; among them, 59 (55.7%) were affected by rectal or sigmoid cancer. The demographic, anthropometric, and clinical data of patients with and without AL are summarized in Table 1. Patients with AL had a significantly lower mean BMI value (23.5 ± 4.2 vs 25.2 ± 4.1, *p* = 0.0002) and a significantly higher mean Charlson comorbidity index (6.5 ± 3.0 vs 5.5 ± 2.3, *p* = 0.0017). Furthermore, there was a significantly higher percentage of TNM stage III patients (51.9% vs 35.5%, *p* = 0.0368) in the group of patients with AL in comparison to those without; the latter had significantly more patients of TNM stage II (33.9% vs 19.8%, *p* = 0.0353) and significantly less pathological grade 3 tumors (13.7% vs 27.4%, *p* = 0.0027). AL was treated with a surgical operation in most cases (72.6%) and without surgery in the remaining cases. AL patients had significantly higher percentage of concomitant complications (50% vs 17.1%, *p* = 0.0042), a greater length of stay (24.1 ± 17.3 vs 10.6 ± 4.4, *p* < 0,0001), and significantly worse 30-day postoperative mortality (10.4% vs 0.5%, *p* < 0,0001).

**Table 1:** The main demographic, clinical, and surgical features of patients with and without anastomotic leakage are presented

	All patients 1432	Leakage 106	No leakage 1326	***p*** value
**Male sex,*n*(%)**	822 (57.4)	65 (56.6)	757 (57.1)	ns
**Age (mean ± SD), years**	65.8 ± 13.7	67.4 ± 11.4	65.7 ± 13.9	ns
**BMI (mean ± SD)**	25.1 ± 4.2	23.5 ± 4.2	25.2 ± 4.1	0.0002
**ASA score (mean ± SD)**	2.2 ± 0.8	2.2 ± 0.8	2.2 ± 0.8	ns
**Charlson comorbidity index (mean ± SD)**	5.5 ± 2.3	6.5 ± 3.0	5.4 ± 2.3	0.0017
**Disease localization**				
**Right colon,*n*(%)**	451 (31.5)	23 (21.7)	428 (32.3)	ns
**Transverse colon,*n*(%)**	99 (6.9)	7 (6.6)	92 (6.9)	ns
**Left colon,*n*(%)**	149 (10.4)	15 (14.1)	134 (10.1)	ns
**Sigmoid,*n*(%)**	331 (23.1)	19 (17.9)	312 (23.5)	ns
**Rectum,*n*(%)**	395 (27.6)	40 (37.7)	355 (26.8)	ns
**Multiple,*n*(%)**	7 (0.5)	2 (1.9)	5 (0.4)	ns
**AJCC stage**				
**0,*n*(%)**	13 (0.9)	2 (1.9)	11 (0.8)	ns
**I,*n*(%)**	242 (16.9)	16 (15.1)	226 (17)	ns
**II,*n*(%)**	470 (32.8)	21 (19.8)	449 (33.9)	0.0353
**III,*n*(%)**	526 (36.7)	55 (51.9)	471 (35.5)	0.0368
**IV,*n*(%)**	167 (11.7)	12 (11.3)	155 (11.7)	ns
**NA,*n*(%)**	14 (1)	0 (0)	14 (1.1)	ns
**Tumor grALing**				
**G1,*n*(%)**	193 (13.5)	9 (8.5)	184 (13.9)	ns
**G2,*n*(%)**	1018 (71.1)	68 (64.1)	950 (71.6)	ns
**G3,*n*(%)**	211 (14.7)	29 (27.4)	182 (13.7)	0.0027
**NA,*n*(%)**	10 (0.7)	0 (0)	10 (0.7)	ns
**Distance from AV, rectal tumors (mean ± SD), cm**	9.5 ± 3.3	9.2 ± 3.3	9.5 ± 3.3	ns
**Resection type (total procedures)**	1438	106	1332	
**Right hemicolectomy,*n*(%)**	474 (33)	21 (19.8)	453 (34)	0.0337
**Left hemicolectomy,*n*(%)**	151 (10.5)	18 (17)	133 (10)	ns
**Anterior resection,*n*(%)**	566 (39.4)	56 (52.8)	510 (38.3)	ns
**Trasverse resection,*n*(%)**	197 (13.7)	7 (6.6)	190 (14.3)	ns
**Total colectomy,*n*(%)**	25 (1.7)	4 (3.8)	21 (1.6)	ns
**Other,*n*(%)**	25 (1.7)	0 (0)	25 (1.9)	ns
**Surgical approach**				
**Open,*n*(%)**	1060 (74)	70 (66)	990 (74.7)	ns
**Laparoscopic,*n*(%)**	372 (26)	36 (34)	336 (25.3)	ns
**Leakage postoperative day, median (95% CI)**	–	6 (5–7)	–	–
**Leakage treatment**				
**Open,*n*(%)**	–	77 (72.6)	–	–
**Conservative,*n*(%)**	–	25 (23.6)	–	–
**NA,*n*(%)**	–	4 (3.8)	–	–
**Other complications,*n*(%)**	1152 (80.4)	53 (50)	227 (17.1)	0.0042
**Length of stay, (mean ± SD), days**	11.7 ± 7.5	24.1 ± 17.3	10.6 ± 4.4	< 0,0001
**30-day mortality**	18 (1.3)	11(10.4)	7 (0.5)	< 0,0001

*AJCC* American Joint Committee on Cancer, *ASA* American Society of Anesthesiology, *AV* anal verge, *BMI* body mass index, *CI* confidence interval, *F* females, *M* males, *NA* not available, *ns*: not significant, *SD* standard deviation. Statistical significance at 0.05

As shown by Table 2, no significant differences between patients with and without AL were found in the median values of white blood cells, neutrophils, monocytes, lymphocytes, and platelets before surgery; similarly, the red cell distribution width (RDW), as well as cell ratios were not statistically different, with the only exception of PLR (200 vs 178, *p* = 0.038) which showed a limited but statistically significant difference. On the first postoperative day, some further differences in blood cell populations and indexes were observed, but the greatest statistical differences were registered on the 4th postoperative day. On that day, patients with AL had significantly greater mean WBC (10 vs 7.9, *p* < 0.0001) and neutrophil (8 vs 5.7, *p* < 0.0001) values, but lower mean lymphocyte (0.9 vs 1.10, *p* < 0.0001) values. In addition, the mean NLR (9.6 vs 5.3, *p* < 0.0001), dNLR (4.7 vs 2.9, *p* < 0.0001), and PLR (254 vs 218, *p* < 0.0001) values were consistently greater in patients who developed an AL (Table 2). In a multiple regression analysis model including the most impacting risk factors on AL and the indexes with statistically significant differences between patients with and without AL in the 4th postoperative day, the BMI (OR 0.881, 95% CI 0.832–0.948, *p* < 0.001), Charlson comorbidity index (OR 1.278, 95% CI 1.152–1.417, *p* < 0.001), and 4th postoperative day NLR (OR 1.068, 95% CI 1.021–1.117, *p* = 0,004) were shown to be independent factors associated with AL.

**Table 2:** Comparisons of the median values of the indexes studied preoperatively and at the 1st and 4th postoperative days, in patients with and without anastomotic leakage

Parameter	Patients	Preoperatively	1st postoperative day	4th postoperative day
WBC median (IQR)	Non-AL	6.50 (5.40–8.30)	10.40 (8.30–12.90)	7.90 (6.30–9.90)
	AL	6.25 (5.20–7.60)	9.10 (7.30–12.10)	10.00 (7.65–12.72)
	***p value***	***ns***	***ns***	***< 0.0001***
Neutrophils median (IQR)	Non-AL	4.10 (3.20–5.50)	8.40 (6.50–10.70)	5.70, 4.40–7.52
	AL	4.00 (3.10–5.03)	7.50 (6.05–10.10)	8.00, 5.92–10.10
	***p value***	***ns***	***ns***	***0.0001***
Monocytes median (IQR)	Non-AL	0.50 (0.40–0.60)	0.60 (0.50–0.90)	0.50 (0.40–0.70)
	AL	0.50 (0.40–0.60)	0.50 (0.30–0.60)	0.50 (0.40–0.60)
	***p value***	***ns***	***< 0.0001***	***ns***
Lymphocytes median (IQR)	Non-AL	1.50 (1.10–1.90)	1.00 (0.70–1.40)	1.10 (0.80–1.40)
	AL	1.30 (1.00–1.82)	0.80 (0.60–1.10)	0.90 (0.70–1.17)
	***p value***	***ns***	***0.0002***	***< 0.0001***
Platelets median (IQR)	Non-AL	258 (211–319)	220 (179–271)	231 (184–285)
	AL	267 (216–323)	216 (175–263)	240 (182–306)
	***p value***	***ns***	***ns***	***ns***
RDWmedian (IQR)	Non-AL	14.8 (13.8–16.6)	14.9 (13.8–16.8)	15.0 (13.8–17.0)
	AL	15.1 (13.7–17.2)	16.0 (13.7–17.5)	15.9(13.9–17.6)
	***p value***	***ns***	***ns***	***ns***
	AL	8.0 (6.3–13.8)	31.5 (28.0–45.0)	105.0 (82.0–132.0)
	***p value***	***ns***	***ns***	***< 0.0001***
NLR median (IQR)	Non-AL	2.90 (2.10–3.90)	8.35 (6.00–11.80)	5.30 (3.60–7.40)
	AL	3.30 (2.28–4.13)	9.80 (7.12–12.30)	9.60 (6.55–10.98)
	***p value***	***ns***	***0.007***	***< 0.0001***
dNLR median (IQR)	Non-AL	1.90 (1.50–2.40)	4.60 (3.58–6.10)	2.90 (2.10–3.90)
	AL	2.10 (1.60–2.50)	5.00 (4.10–6.22)	4.70 (3.40–5.50)
	***p value***	***ns***	***0.025***	***< 0.0001***
LMR median (IQR)	Non-AL	3.00 (2.20–4.00)	1.60 (1.10–2.10)	2.00 (1.50–2.80)
	AL	2.70 (2.20–4.10)	1.80 (1.30–2.18)	2.00 (1.40–2.40)
	***p value***	***ns***	***ns***	***ns***
PLR median (IQR)	Non-AL	178 (129–253)	230 (158–317)	218 (154–288)
	AL	200 (141–276)	270 (190–374)	254 (212–338)
	***p value***	***0.038***	***0.0009***	***< 0.0001***

*AL* anastomotic leakage, *IQR* interquartile range, *LMR* lymphocyte to monocyte ratio, *NLR* neutrophil to lymphocyte ratio, *PLR* platelet to lymphocyte ratio, *RDW* red blood cell distribution width, *ns* not significant, *WBC* white blood cells. Statistical significance at 0.05

**Fig. 1:**
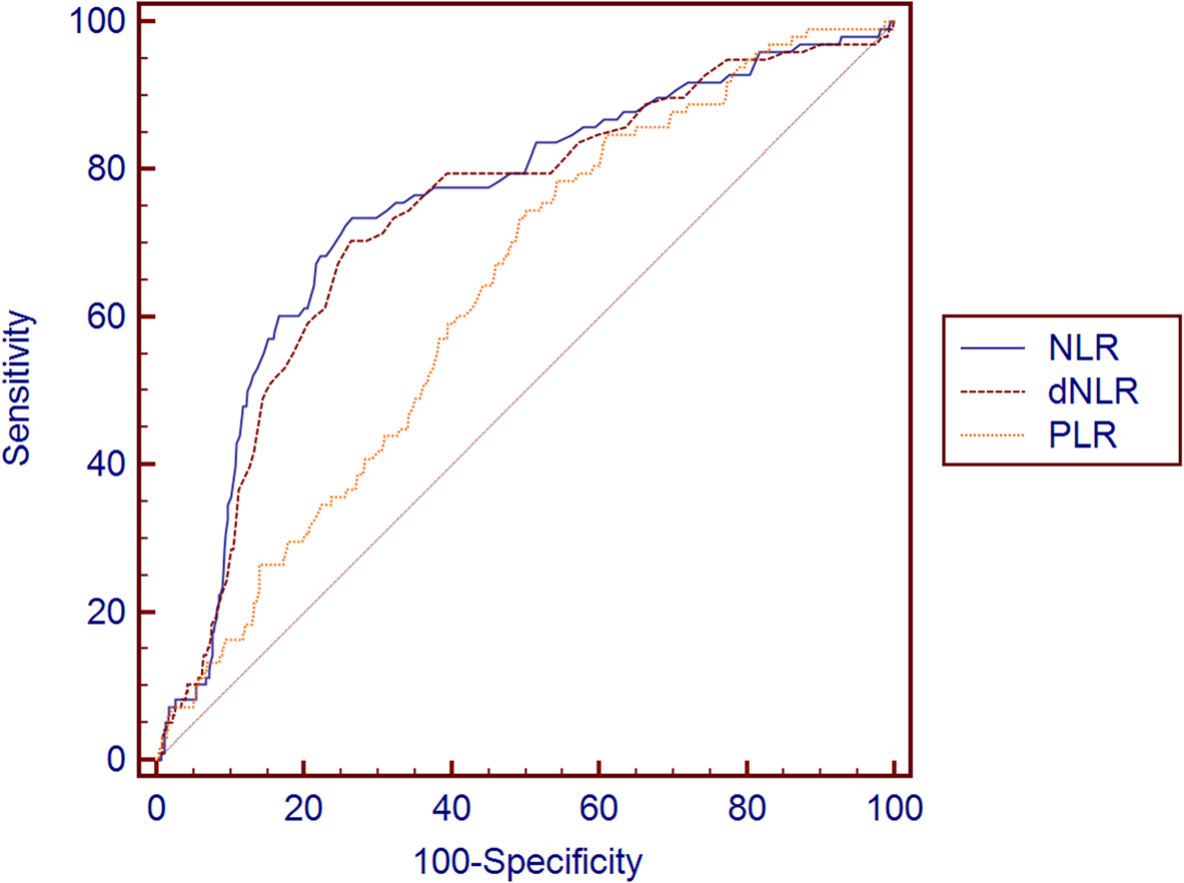
ROC curves of NLR, dNLR, and PLR in predicting anastomotic leakage

We performed ROC curve analysis for the indexes which showed statistically significant differences between the two groups of patients on the 4th postoperative day (Table 3, Fig. 1). NLR at a cutoff point of 7.1 showed the best AUC (0.744, 95% CI 0.719–0.768) with a sensitivity and specificity of 72.7% and 73.4%, respectively, followed by the dNLR (0.732, 95% CI 0.707–0.757) at a cutoff point of 3.8. PLR showed a poor result in ROC analysis.

**Table 3:** ROC curves of the indexes under evaluation as predictive markers of anastomotic leakage

Marker	AUC	95% CI	***p*** value	Cutoff	Sensitivity	Specificity
**NLR**	0.744	0.719–0.768	< 0.0001	> 7.1	72.73	73.44
**dNLR**	0.732	0.707–0.757	< 0.0001	> 3.8	69.70	73.70
**PLR**	0.632	0.605–0.659	< 0.0001	> 217	74.49	49.87

*AUC* area under the curve, *CI* confidence interval, *NLR* neutrophil to lymphocyte ratio, *dNLR* derived NLR, *PLR* platelet to lymphocyte ratio. Significance at 0.05

## Discussion

Anastomotic leakage is one of the most serious postoperative adverse events in colorectal surgery [1–6]. This was confirmed also in the present study considering the rate of postoperative concomitant complications, length of stay, and 30-day mortality, which were significantly higher in patients with AL than in those with an uncomplicated postoperative course. The rate of AL in our series (7.4%) was similar to that reported in other recent articles [10, 15]. AL patients in our study had lower mean BMI values in comparison to those without AL, and BMI was found to be an independent factor influencing AL in multivariate analysis; this result is somewhat unexpected, considering that obesity is traditionally considered one of the main risk factors of AL [21]. On the other hand, these patients had a worse Charlson comorbidity index, as well as higher tumor TNM stage and histological grade. This may be associated with a greater level of systemic inflammation, which in one hand determines the increased AL rates, and on the other alters several hematological biomarkers, like those investigated herein. Charlson comorbidity index and NLR in the 4th postoperative day (other than BMI) were shown to be independent factors associated with AL in a multivariate analysis model including also age, sex, ASA score, TNM stage of the tumors, and 4th postoperative day RDW, dNLR, and PLR. No significant correlation between the type of surgery performed (open or laparoscopy) and the occurrence of AL was observed. AL required surgical re-operation in most cases (72.6%); in the remaining cases, it was treated conservatively, mainly through a “wait and see” approach in patients with drain tubes and clinical conditions which permitted the resolution of the leakage without surgery. No statistically significant correlations with NLR values in the 4th postoperative day were found between patients with and without resurgery for AL.

To our knowledge, this is the first study to evaluate the role of the dNLR, LMR, and PLR in predicting AL. These simple blood count indexes, together with the NLR and RDW, have been demonstrated to have a prognostic potential in several chronic pathological conditions, including colorectal cancer and colorectal liver metastases [22–26], and a potential role in predicting outcomes in surgical procedures [15–19, 27–29]. NLR is the most studied index to this purpose. Josse et al. retrospectively investigated its role in predicting complications in 583 patients who underwent surgical resection for suspected or confirmed colorectal cancer [29]. The authors found that a preoperative NLR greater or equal to 2.3 was significantly associated with a major perioperative complication rate; on multivariate analysis, a high NLR and Charlson comorbidity index ≥ 3 were significantly related to major morbidity. Nevertheless, they did not detect any relationship between an elevated preoperative NLR and specific complication types, although there was a trend towards higher NLR values in patients with AL [29].

Miyakita et al. published a study on 260 patients with rectal cancer who underwent radical surgery to examine the relations between complications and 5 types of risk scores, including the preoperative NLR [15]. Complications developed in 56 patients (21.5%), and 18 patients with AL were encountered. The authors evidenced that the levels of NLR calculated in blood samples obtained at initial presentation and before chemo-radiotherapy were significantly associated with surgical complications in general, and especially to AL. In particular, they established that a preoperative NLR cutoff point at 2.21 was an independent predictor of AL (*p* = 0.0089, odds ratio = 8.24) and that the sensitivity and specificity of the test at this cutoff point was 83% and 47%, respectively [15]. This cutoff value seems very low to invest a clinical role in detecting AL, and was lower from the median values observed in both patients with and without AL in our cohort; in addition, we did not detect any statistically significant difference in the preoperative values of NLR between the two groups of patients.

Another study recently published by Mik et al. included 724 patients who underwent elective open colorectal surgery, and (among them) the rate of AL was 4.6% [10]. In this study, blood samples were obtained also on the 1st and 4th postoperative days, and both CRP and NLR were evaluated. The authors found a statistically significant difference in the mean value of NLR on the 4th postoperative day, between patients with (9.03 ± 4.13) and without (4.45 ± 2.25) AL (*p* = 0.0012). The ROC analysis showed a sensitivity of 69% (95% CI, 65–73), and a specificity of 78% (95% CI, 74–82) at a cutoff point of 6.5, with an AUC of 0.68 [10]. In our cohort, the AUC was greater (0.744), the sensitivity was slightly higher (73%), and the specificity lower (73%) at an NLR cutoff point of 7.1 on postoperative day 4. This value was relatively close to the NLR cutoff value in patients with AL found in the study of Mik et al.; in addition, the median NLR value found in our series (9.60) was close to the median value found by Mik et al. (9.03) in this subset of patients.

More recently, Walker et al. published their results on the predictive roles of CRP, PCT, and NLR on 136 patients retrospectively enrolled (eleven AL patients, 8.1%) [30]. Median CRP values were found to be significantly higher in patients with AL in comparison to those with an uncomplicated course on postoperative days 2 through 5. PCT median value differences never reached statistical significance within the same time frame, while those of NLR were significantly higher in AL patients on postoperative days 3 and 4. ROC analysis evidenced that the cutoff for CRP (105 mg/L) with the highest sensitivity (100%) and specificity (56.5%) was on postoperative day 5; this figure is consistently lower than those reported by Mik et al. The NLR showed the best predictive ability at a cutoff value of 6.15 in day 4 (sensitivity of 100% and specificity of 61.8%), as observed also in our cohort. Another recent study, performed in 44 case-matched patients, reported a poor AUC (0.697) in predicting AL at an NLR cutoff value of 8.7, with sensitivity and specificity of 52% and 88%, respectively [31].

The RDW and the LMR did not show any predictive ability for the early detection of AL in our study. To this regard, a retrospective pilot study by Paliogiannis et al. investigated the role of the preoperative RDW and mean platelet volume (MPV) in 42 case-matched patients who underwent oncological colorectal surgery [32]. The authors found higher mean values for both indexes in patients with than without AL, but in multiple regression analysis only, the RDW remained significantly associated to the AL, and the AUC reported was poor (0.673, cutoff 11%, sensitivity 90.2%, and specificity 38.1%). In the present study, the RDW did not show any predictive ability neither before nor after surgery. As opposed, the PLR was significantly higher in AL patients in all the evaluations performed, with the statistical significance increasing from the preoperative to the 4th postoperative day. Also in this case, the AUC on the 4th postoperative day was poor, but the finding deserves further evaluation in future studies.

Our study has some limitations, mainly the retrospective design, the lack of information about treatments that may alter the indexes under evaluation (like steroids), as well as potential variability in the perioperative management of the patients and laboratory testing. On the other hand, it includes the largest cohort employed so far for the study of the role of specific inflammatory indexes in predicting AL, some of them tested for the first time. Further prospectively designed trials are necessary to investigate the prognostic effect and the clinical applicability of NLR in patients who develop AL following colorectal resection.

## Conclusions

Among the blood cell indexes of systemic inflammation investigated in this study, NLR evaluated on the 4th postoperative day showed the better results in predicting AL. Therefore, NLR which is a simple, inexpensive, and widely available index, might be a useful tool in clinical practice in predicting the occurrence of AL in patients undergoing elective colorectal surgery. Nevertheless, its potential usefulness in daily practice needs to be further evaluated in future prospective studies.

## Data Availability

The datasets used and/or analyzed during the current study are available from the corresponding author on reasonable request.

## Abbreviations

AJCC: American Joint Committee on Cancer
AL: Anastomotic leakage
ASA: American Society of Anesthesiologists
BMI: Body mass index
CRP: C-reactive protein
LMR: Lymphocyte to monocyte
NLR: Neutrophil to lymphocyte
PCT: Procalcitonin
PLR: Platelet to lymphocyte
RDW: Red blood cell distribution width
